# Visual acuity, contrast sensitivity, and quality of life after bilateral implantation of multifocal diffractive intraocular lens

**DOI:** 10.5935/0004-2749.20230054

**Published:** 2023

**Authors:** Marjorie F. N Queiroz, Felipe Q. T Ferreira, Gilberto Shimoda, Carlos Roberto Padovani, Antonio C Lottelli

**Affiliations:** 1 Division of Ophthalmology, Surgical Specialties and Anesthesiology Department, Faculdade de Medicina de Botucatu, Universidade Estadual Paulista “Júlio de Mesquita Filho, Botucatu, SP, Brazil.; 2 Centro de Microcirurgia Ocular Atibaia, Atibaia, SP, Brazil.; 3 Bioscience Institute, Universidade Estadual Paulista “Júlio de Mesquita Filho, Botucatu, SP, Brazil.

**Keywords:** Visual acuity, Quality of life, Patient satisfaction, Lens implantation, intraocular, Unified Health System, Acuidade visual, Qualidade de vida, Satisfação do paciente, Implante de lente intraocular, Sistema Único de Saúde

## Abstract

**Purpose:**

To evaluate visual outcomes, satisfaction, and quality of life of patients assisted in a Medical School hospital by the Brazilian Public Health System, who underwent bilateral diffractive multifocal intraocular lens implantation.

**Methods:**

Case series study with intervention, including 20 patients who underwent bilateral implantation of multifocal IOL EyeDiff^®^ (Eyeol UK, Dunstable, UK). Exclusion criteria were corneal astigmatism >1.5 cylindrical diopters, previous ocular surgery or ocular disease, and intra- or postoperative complications. Patients were evaluated one, three, and six months after surgery. Monocular and binocular visual acuity for distance, intermediate and near, under photopic and mesopic conditions, monocular contrast sensitivity under photopic conditions, defocus curve, and quality of life were assessed.

**Results:**

Monocular distance-corrected visual acuity was 0.3 logMAR or better and monocular distance-corrected near visual acuity was J3 or better in all eyes under photopic conditions. Binocular distance-corrected near visual acuity was J1 in all cases. Contrast sensitivity was at the minimum level of normality for low and high spatial frequencies and within normal limits for intermediate spatial frequency. The quality of life questionnaire showed a high level of patient satisfaction.

**Conclusion:**

Bilateral implantation of the multifocal intraocular lens EyeDiff^®^ provides patients with good visual acuity and quality of life, besides spectacle independence. The visual acuity and contrast sensitivity progressively improved between one and six postoperative months.

## Abstract (pt)

**Objetivo:**

Avaliar os resultados visuais, satisfação e qualidade de vida de pacientes atendidos em um hospital escola pelo Sistema Único de Saúde, submetidos a implante bilateral de lente intraocular multifocal difrativa.

**Métodos:**

Estudo tipo série de casos com intervenção, incluindo 20 pacientes submetidos a implante bilateral da lente intraocular multifocal difrativa EyeDiff^®^ (Eyeol UK, Dunstable, UK). Os critérios de exclusão foram astigmatismo corneano >1,5 dioptria cilíndrica, cirurgia ou doença ocular prévias e complicações intraoperatórias ou pós-operatórias. Os pacientes foram avaliados após 1, 3 e 6 meses da cirurgia. Foram avaliadas a acuidade visual monocular e binocular para longe, intermediário e perto sob condições fotópica e mesópica, sensibilidade ao contraste monocular sob condições fotópicas, curva de *defocus* e questionário para avaliação da qualidade de vida.

**Resultados:**

A acuidade visual para longe corrigida monocular foi de 0,3 logMAR ou melhor e a acuidade visual para perto com correção para longe foi J3 ou melhor em todos os olhos, sob condições fotópicas. A acuidade visual binocular para perto com a correção para longe foi J1 em todos os casos. A sensibilidade ao contraste estava no nível mínimo de normalidade para frequências espaciais baixas e altas e abaixo dos limites normais para frequência espacial intermediária. O questionário de qualidade de vida mostrou que os pacientes apresentavam altos níveis de satisfação.

**Conclusão:**

O implante bilateral da lente intraocular multifocal EyeDiff^®^ proporcionou boa acuidade visual e qualidade de vida, e independência de óculos aos pacientes. A acuidade visual e a sensibilidade ao contraste melhoraram progressivamente entre um e seis meses de pós-operatório.

## INTRODUCTION

The evolution of cataract surgery has improved predictability of outcomes and visual acuity (VA) recovery in a short postoperative period^(1-4)^, and partially, this is related to the increasing number of surgeries with a small incision^(5)^. Additionally, the evolution of technology in the development of intraocular lenses (IOLs) allowed greater spectacle independence after surgery^(1-4)^.

Modern IOLs do not just solve aphakia but can also reduce ocular aberrations, protect the retina against ultraviolet light, and improve near, intermediate, and distance VA^(6)^.

Multifocal IOLs, introduced in the 1980s^(7)^, were developed to improve the quality of patients’ life who underwent cataract surgery by improving acuity and visual function, which can lead to greater spectacle independence^(8)^. However, undesirable symptoms, such as halos and glare, may occur^(9)^, which can lead to difficulties in performing tasks, such as driving at night and reading in poorly lit environments^(10)^. Although multifocal IOLs have better intermediate vision than monofocal IOLs^(10)^, they are still inferior to near and distance VA^(11)^.

This study aimed to evaluate postoperative visual outcomes and the quality of patients’ life who underwent bilateral multifocal IOL implantation and were followed up in a Medical School hospital of the Brazilian Public Health System.

## METHODS

This was a case series study with intervention carried out at Botucatu Medical School from *Universidade Estadual Paulista* (UNESP), São Paulo, Brazil. The study was approved by the local ethics committee. Before the procedure, patients signed a consent form.

The inclusion criteria were age over 50 years and bilateral senile cataract. The exclusion criteria were corneal astigmatism greater than 1.5 diopters (D), amblyopia, history of previous intraocular surgery or ocular disease, lack of motivation to perform surgical procedure bilaterally, inability to understand and collaborate in performing the exams, refusal to sign the consent form, and intra- or postoperative complications. The patients underwent bilateral phacoemulsification with multifocal IOL implantation, with a minimum interval of 7 days between the first and second eye treatment.

### Preoperative evaluation

General characteristics, such as age and gender, were analyzed. Patients were evaluated for distance VA at four meters (m), intermediate VA at 60 centimeters (cm), and for near VA at 33 cm with and without optical correction, under photopic conditions at 85 candelas per square meter (cd/m^2^), and under mesopic conditions at 3 cd/m^2^. For distance, the Early Treatment Diabetic Retinopathy Study chart and the logarithm of the minimum angle of resolution (logMAR) were used. Jaeger chart was used for near and intermediate VA. Additionally, biomicroscopy, intraocular pressure (IOP) measurement, and fundus biomicroscopy were performed.

As complementary examinations, biometry (IOL Master 500^®^, Carl Zeiss Meditec Company, Jena, Germany), and a contrast sensitivity test were performed. The latter was a monocular test, at a distance of 40 cm, using the printed version of the Functional Acuity Contrast Test (F.A.C.T.)^®^ chart (Stereo Optical Company, Chicago, IL, USA) under photopic conditions at 85 cd/m^2^. In this test, each contrast step corresponds to 0.15 log units that represent a loss in contrast of 50% for two contrast steps increase, and the tested spatial frequencies were 1.5, 3, 6, 12, and 18 cycles per degree (cpd).

### Surgical technique

Standard phacoemulsification was performed in all patients by the same surgeon (MFNQ) and a single-piece hydrophilic diffractive multifocal lens with the addition of +3.50 D in the IOL plane (EyeDiff^®^, EyeOL UK Limited, Dunstable, UK) was implanted in the capsular bag.

In the postoperative period, patients were advised to use prednisolone acetate 1% and gatifloxacin 0.3% eye drops six times a day for one week, and prednisolone acetate 1% eye drops four times a day for the subsequent three weeks.

### Postoperative evaluation

At one, three, and six months after second eye surgery, monocular and binocular uncorrected distance (UDVA), intermediate (UIVA), and near (UNVA) VA, corrected distance visual acuity (CDVA), and distance-corrected intermediate (DCIVA) and near (DCNVA) VA were evaluated under photopic conditions. Monocular VA was also assessed, but only monocular and corrected VA were tested in this condition. The contrast sensitivity test was repeated at one and six months postoperatively. Monocular distance VA on the defocus curve was assessed after a 0.50 D increase over the best distance correction, ranging from-3.50 to +3.00 D, after 6 months. VA values were registered for each vergence and evaluated in a two-dimensional graph using a coordinate system in the Cartesian plane. A validated questionnaire based on the National Eye Institute-Visual Functional Questionnaire (NEI VFQ 25) was used in months one and six to assess the quality of life. Data were included in the Excel table and their confidentiality was assured.

### Statistical analysis

For comparison of evaluation times, the analysis of variance was used for the model of repeated measures involving parametric procedure, when the variable in the study was shown to be adherent to the normal distribution of probabilities. Otherwise, the procedure was non-parametric. When the procedure used was parametric, the analysis was complemented with the Bonferroni multiple comparison test and, in the non-parametric case, with the Dunn procedure. For the study of the NEI VFQ-25 questionnaire subdomains, Student’s t-test was used for paired samples^(12)^. Statistical significance was assumed by p-value <0.05.

## RESULTS

**Figure 1:**
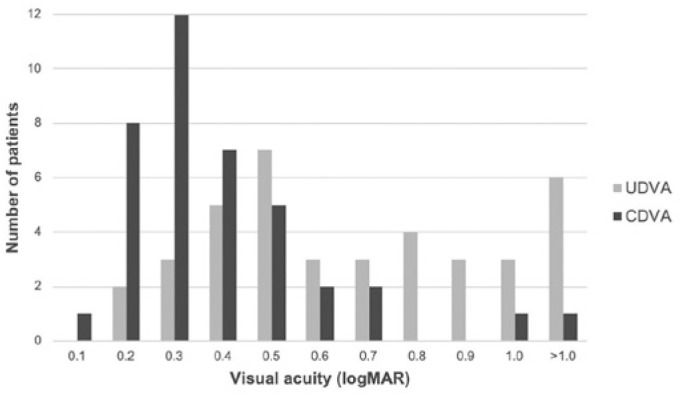
Preoperative uncorrected and corrected distance visual acuity.

Forty-four eyes of 23 patients were operated. Two patients had intraoperative complications in the first operated eye and one in the second eye. These patients were excluded. Thus, 20 patients (40 eyes) were included and analyzed. Eighteen patients (90%) were women. The mean age was 67.5 ± 6.74 years, and the range was from 54 to 79 years. Preoperative distance VA is detailed in figure 1.

UDVA= uncorrected distance visual acuity; CDVA= corrected distance visual acuity.

Postoperative spherical equivalent (SE) after six months was +0.075 D ± 0.475. Postoperative photopic and mesopic distance VA results are described in table 1. Regarding monocular distance VA, under photopic conditions, it was observed that 38 (95%) and 40 (100%) eyes had UDVA of 0.3 logMAR or better after three and six months, respectively. Under mesopic conditions, 17 (42.5%), 21 (52.5%), and 28 (70%) eyes had CDVA of 0.3 logMAR or better after one, three, and six months, respectively. There was a progressive statistically significant improvement in VA in these conditions. A progressive improvement in binocular and photopic VA was also observed, as illustrated in Table 1, but it was not significant.

**Table 1:** Postoperative photopic and mesopic distance visual acuity (logMAR)

	1 month	3 months	6 months	p-value
**Monocular Photopic VA**				
UDVA	0.26 ± 0.12	0.22 ± 0.09	0.21 ± 0.09	0.004
	(0.1 - 0.6)	(0.1 - 0.5)	(0.1 - 0.4)	
CDVA	0.16 ± 0.09	0.14 ± 0.06	0.12 ± 0.05	0.009
	(0 - 0.5)	(0 - 0.3)	(0.1 - 0.3)	
**Binocular Photopic VA**				
UDVA	0.19 ± 0.07	0.16 ± 0.06	0.17 ± 0.06	0.275
	(0.1 - 0.3)	(0.1 - 0.2)	(0.1 - 0.3)	
CDVA	0.13 ± 0.06	0.12 ± 0.05	0.1 ± 0.03	0.082
	(0 - 0.2)	(0 - 0.2)	(0 - 0.2)	
**Mesopic VA**				
CDVA	0.4 ± 0.12	0.36 ± 0.12	0.33 ± 0.1	0.002
	(0.2 - 0.7)	(0.2 - 0.7)	(0.2 - 0.7)	

UDVA= uncorrected distance visual acuity; CDVA= corrected distance visual acuity.

**Figure 2:**
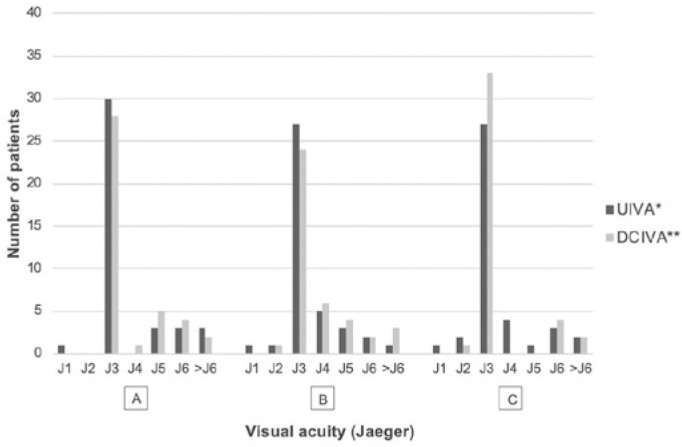
Postoperative monocular and photopic intermediate visual acuity.

**Figure 3:**
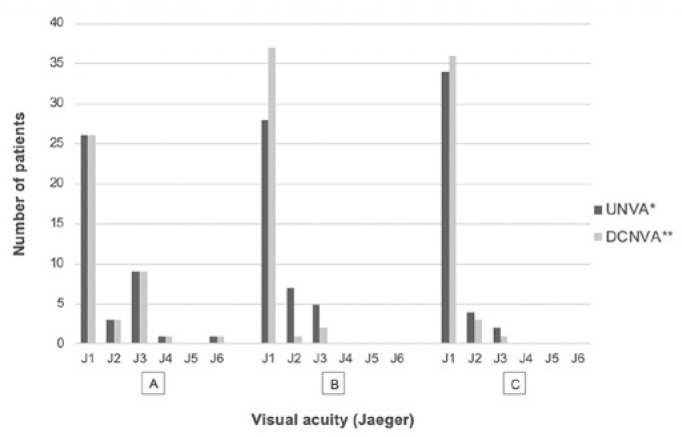
Postoperative monocular and photopic near visual acuity.

Postoperative monocular and photopic intermediate VA are demonstrated in figure 2. Thirty eyes (75%) presented with UIVA of J3 or better after 6 months under photopic conditions. There was no statistical significance when the three visits were compared. Regarding near VA (Figure 3), 40 (100%) eyes had monocular UNVA of J3 or better under photopic conditions after six months, and 19 (95%) patients presented with binocular UNVA of J1. In 100% of the patients, binocular DCNVA was J1 under photopic conditions after six months of surgery. There was a progressive significant improvement of VA during the follow-up (p<0.01). Under mesopic conditions, DCNVA was J3 or better in 22 (55%), 26 (65%), and 34 (85%) eyes after one, three, and six months, respectively, and this improvement was statistically significant (p<0.01).

UIVA= uncorrected intermediate visual acuity; DCIVA= distance-corrected intermediate visual acuity. A= 1 month; B= 3 months; C= 6 months; *p=0.896; **p=0.172.

UNVA= uncorrected near visual acuity; DCNVA= distance-corrected near visual acuity. A= 1 month; B= 3 months; C= 6 months; *p=0.005; **p=0.001.

**Figure 4:**
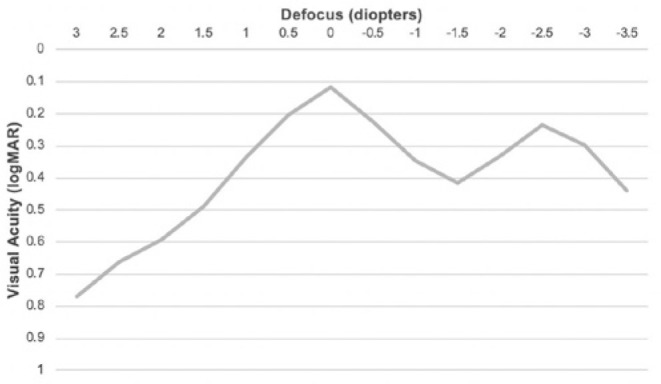
Postoperative defocus curve.

Defocus curve showed two peaks of best VA in vergences 0 and-2.50 D, where the mean VA was 0.12 ± 0.045 logMAR and 0.23 ± 0.11 logMAR at month six, respectively (Figure 4).

**Figure 5:**
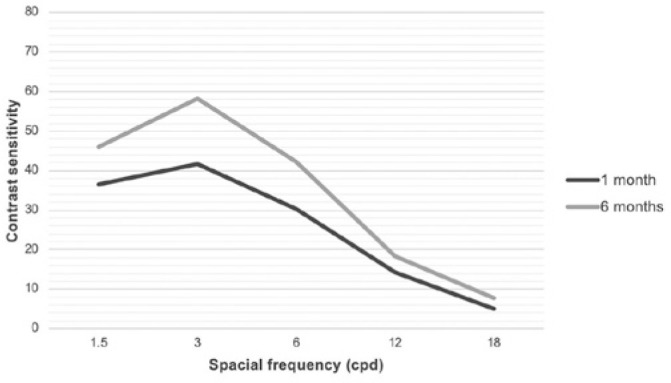
Postoperative contrast sensitivity test with distance correction.

Postoperative contrast sensitivity outcomes with distance correction are shown in figure 5. Values were below normal limits in both evaluations at spatial frequencies of 6 and 12 cpd. A statistically significant improvement in all spatial frequencies was observed when comparing the values at months one and six (p<0.01).

cpd= cycles per degree. *p*<0.05 in all spatial frequencies.

**Figure 6:**
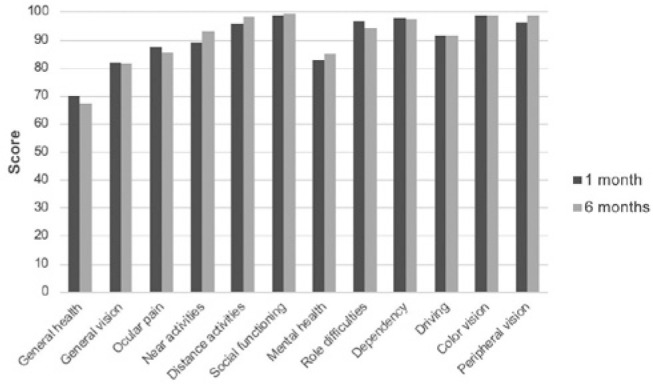
Postoperative quality of life questionnaire results.

The quality of life assessment questionnaire was applied according to its subdomains: general health, general vision, eye pain, near activities, distance activities, social aspects, mental health, activities of daily living, dependence, color vision, and peripheral vision. Overall mean scores of 90.66 and 91 points were obtained at months one and six, respectively, without significant difference (Figure 6).

## DISCUSSION

The present study showed a significant and progressive improvement in monocular UDVA, CDVA, mesopic monocular CDVA, near VA under photopic and mesopic conditions, and contrast sensitivity between the first and six postoperative months. The patients also achieved spectacle independence, as described in the literature on patients who underwent multifocal IOL implant^(5)^.

A study with Tecnis^®^ IOL with different add power reported higher UDVA values of 0.045 ± 0.04 and 0.067 ± 0.068 logMAR^(12)^ and in another study using AcriLISA^®^ IOL, UDVA was-0.05 ± 0.1 logMAR^(13)^. A meta-analysis reported mean UDVA of 0.11 logMAR ± 0.003^(11)^. The mean CDVA, which in the present study was 0.12 ± 0.045 logMAR, was slightly worse compared to that of others: 0.02 ± 0.05 logMAR with Acri.LISA^(14)^ and 0.007 ± 0 logMAR with ReSTOR^®^^(14)^.

A meta-analysis concluded that 99.9% of patients had binocular UDVA of 0.3 logMAR or better after bilateral multifocal IOL implantation, a value similar to that found in the present study, where 100% of the patients presented with this VA value^(11)^.

Many studies show similar results of UDVA and CDVA^(13-15)^, compared to the present study. This is obviously related to the postoperative refractive error. The fact that eyes with keratometric astigmatism up to 1.50 D have been included can explain this finding, as the final SE shows that there was good biometric predictability.

VA may be influenced by retinal sensitivity that tends to decrease in elderly patients^(11)^. Additionally, different measurement methods between studies, different intellectual levels of the patients, and even different IOLs optic quality may explain the small differences found in the CDVA when compared with the literature, but because the present study is a case series, it is not possible to draw any conclusions about it.

Regarding UIVA, 75% of the eyes reached VA of J3 or better, which is considered relatively good for intermediate distance. Data from the literature show that improvement in intermediate VA may occur, even though it was inferior to distance and near VA. A previous study found VA of J3 or better for intermediate VA in 83% of the cases after implantation of diffractive multifocal IOL with the addition of +3.0 D^(8)^.

All patients presented binocular DCNVA of J1 under photopic conditions after 3 months of surgery. This demonstrates good performance of the IOL for near VA without refractive error.

The worsening of visual function under mesopic conditions has already been described, even in young and healthy patients^(16)^. It is compatible with the outcomes observed in the present study, where patients presented worse performance under mesopic conditions compared to photopic conditions. A progressive significant improvement of distance and near VA under mesopic conditions in 6 months was also observed. Although many prospective studies assess VA in more than one visit, the results observed in the last visit are chosen to be analyzed. Therefore, we have not found any study with documented mesopic VA tested with 100% contrast at different periods to compare this progressive improvement. This finding is probably related to the process of neuroadaptation.

Concerning the defocus curve, there was a second peak of better VA in the vergence of-2.50 D. This demonstrates that the addition power for near VA in the EyeDiff^®^ IOL is close to +2.50 D in the spectacle plane, which gives the patient better near VA around 40 cm. Patients included in this study had near VA tested at a fixed distance of 33 cm and this may have influenced the results.

Although values presented below normal limits for medium spatial frequencies and at the lower limit for other spatial frequencies, contrast sensitivity improved significantly from one to six months when the two time points were compared. One study tested contrast sensitivity in the medium spatial frequency in the presence of glare and observed that there was a decrease in contrast threshold after 6 months^(17)^. Previous studies with other multifocal IOLs have not had similar findings^(7,10,12)^, and further studies should be performed to confirm and understand these results. The relationship between the implantation of multifocal IOL and the reduction of contrast sensitivity in the postoperative period has already been described^(18,19)^, and this can be partly explained by the division of light that occurs to create two or more images^(11)^. There are, however, studies where contrast sensitivity was similar when comparing postoperative results of multifocal and monofocal IOLs^(10)^. A meta-analysis that included studies comparing results of multifocal IOLs with monofocal IOLs found that in two-thirds of them, where there was a difference between groups, the results of multifocal IOLs were lower at high spatial frequencies^(11)^. It is known that contrast sensitivity may also be reduced under mesopic conditions^(13,20)^, so we believe that future studies are needed to analyze contrast sensitivity after implantation of EyeDiff IOL^®^ under mesopic conditions with and without glare. Contrast sensitivity was assessed monocularly in this study, which may have influenced analysis, since the assessment was binocular in most studies. Moreover, it has been shown that low-contrast distance VA is better during binocular testing compared to monocular^(21)^.

In the present study, the results of the questionnaire for quality of life evaluation showed that the implantation of multifocal IOL was not associated with visual disturbances that alter the quality of life in the postoperative period, as high average scores were obtained for all subdomains. High scores for subdomains, such as near and distance activities, social aspects, activities of daily living, and dependence, illustrate the positive influence of multifocal IOL implantation on their daily life.

Patients included in the study were monitored at the public health service and presented with moderate to advanced cataracts at the time of surgery, and consequently, deteriorated VA, which may be related to the waiting time to reach the treatment. This may have influenced their expectations since, by the time they started the treatment, their aim was to improve VA regardless of the need of wearing glasses.

Undesirable postoperative symptoms, which are mainly glare and optic aberrations, can rarely result in IOL explantation^(22)^. No patients required explantation in this study. In the literature, 0 to 10% of patients complained of disabling halos or glare symptoms and overall satisfaction ranged from 61.8% to 100%^(11)^. Moreover, high expectation and residual refractive error account for about 28% of dissatisfaction causes^(22)^. Posterior capsule opacification, surface ocular disease, and intraoperative complications can also result in dissatisfaction in multifocal implants^(23,24)^.

IOLs with lower additions have lower halos and glare indices, probably due to the smaller number of diffractive steps^(25)^. A previous study observed that patients who underwent IOL implantation with higher addition had a significantly worse general index of satisfaction when assessed by a questionnaire. The same group presented the most complaints of halos and glare vision, although it was not significant^(12)^.

There is a tendency for patients who develop adverse symptoms after implantation of multifocal IOL to become more tolerant to them approximately six months after surgery. There might be a learning effect associated with neural adaptation in the first few months after surgery, causing a reduction in the symptoms^(11)^.

It is believed that neuroadaptation plays an important role in the favorable postoperative results, especially regarding dysphotopsia. Understanding the mechanisms of neuroadaptation may aid in postoperative management, improving the results of multifocal implantation^(17)^.

In general, independence from glasses overcomes the side effects with the use of multifocal IOLs. However, the choice of the IOL should be customized and decided with the patient considering their real motivation^(5)^. It is necessary to evaluate the patient’s lifestyle, including occupational and recreational activities, to choose the best optical correction^(7)^. Good postoperative results depend on the patient’s careful selection, meticulous biometry, and accuracy of the formulas for calculating the dioptric power of IOL^(4)^.

We pointed out the small sample size, which included patients with corneal astigmatism ≥1.0 D as a limitation of this study. Additionally, the use of the Jaeger chart for analysis of intermediate and near VA could have influenced the analysis of the results, as there is no standardization of the chart by the manufacturers^(5)^. Detailed analysis of the cornea with tomography and aberrometry was not included in the preoperative examination, which would be important since irregular astigmatism may lead to worse postoperative visual performance^(26,27)^. Besides that, the questionnaire for quality of life was not accessed on preoperative examination.

In conclusion, bilateral implantation of the multifocal IOL EyeDiff^®^ provides good VA, quality of life, and spectacle independence for the patients. The VA and contrast sensitivity progressively improved from one to six months postoperatively.
